# Fertility Preservation: How to Preserve Ovarian Function in Children, Adolescents and Adults

**DOI:** 10.3390/jcm10225247

**Published:** 2021-11-11

**Authors:** Marie-Madeleine Dolmans, Camille Hossay, Thu Yen Thi Nguyen, Catherine Poirot

**Affiliations:** 1 Gynecology Research Unit, Institut de Recherche Expérimentale et Clinique, Université Catholique de Louvain, Av. Mounier 52, 1200 Brussels, Belgium; camille.hossay@uclouvain.be (C.H.); thu.nguyen@uclouvain.be (T.Y.T.N.); 2 Department of Gynecology, Cliniques Universitaires Saint-Luc, Av. Hippocrate 10, 1200 Brussels, Belgium; 3 Department of Hematology, AYA Unit, Saint Louis Hospital AP-HP, 1 Avenue Claude Vellefaux, 75010 Paris, France; catherine.poirot@aphp.fr; 4 Médecine Sorbonne Université, Site Pitié Salpêtrière, 91 Bd de l’Hôpital, 75013 Paris, France; 5 Department of Reproductive Biology, Cochin Hospital AP-HP, 123 Bd de Port Royal, 75014 Paris, France

**Keywords:** fertility preservation, oocyte vitrification, ovarian tissue cryopreservation, cancer patients, pediatric patients

## Abstract

Chemotherapy, pelvic radiotherapy and ovarian surgery have known gonadotoxic effects that can lead to endocrine dysfunction, cessation of ovarian endocrine activity and early depletion of the ovarian reserve, causing a risk for future fertility problems, even in children. Important determinants of this risk are the patient’s age and ovarian reserve, type of treatment and dose. When the risk of premature ovarian insufficiency is high, fertility preservation strategies must be offered to the patient. Furthermore, fertility preservation may sometimes be needed in conditions other than cancer, such as in non-malignant diseases or in patients seeking fertility preservation for personal reasons. Oocyte and/or embryo vitrification and ovarian tissue cryopreservation are the two methods currently endorsed by the American Society for Reproductive Medicine, yielding encouraging results in terms of pregnancy and live birth rates. The choice of one technique above the other depends mostly on the age and pubertal status of the patient, and personal and medical circumstances. This review focuses on the available fertility preservation techniques, their appropriateness according to patient age and their efficacy in terms of pregnancy and live birth rates.

## 1. Introduction

Gonadotoxic chemotherapy and pelvic irradiation at reproductive age are known to damage the ovaries, which can lead to endocrine dysfunction, cessation of ovarian endocrine activity and early depletion of the ovarian reserve, with the risk of permanent infertility [1]. Gonadal toxicity has also been reported in children [2,3,4].

The risk of premature ovarian insufficiency (POI) depends on factors such as patient age, the existing ovarian reserve and the type of treatment and dose; and it is higher if alkylating drugs or total body irradiation are used [2]. Premature menopause not only negatively impacts fertility potential, but may also seriously affects health and quality of life, with cardiovascular disease, neurodegenerative conditions and osteoporosis posing a threat [5,6,7]. Quality of life after cancer remission, therefore, needs to be urgently addressed, and fertility preservation represents a key challenge in these women [8].

Techniques for fertility preservation have been developed and give these patients genuine hope of becoming mothers when they have overcome their diseases, including embryo cryopreservation, immature or mature oocyte vitrification and ovarian tissue cryopreservation (OTC). A crucial issue at present is that healthcare workers are not fully aware of the remarkable advances happening in fertility preservation research and their implementations in clinical settings [6]. Meeting the rising demand for fertility preservation is indeed challenging [9], and women for whom fertility preservation is needed are growing in number (Table 1).

**Figure 1:**
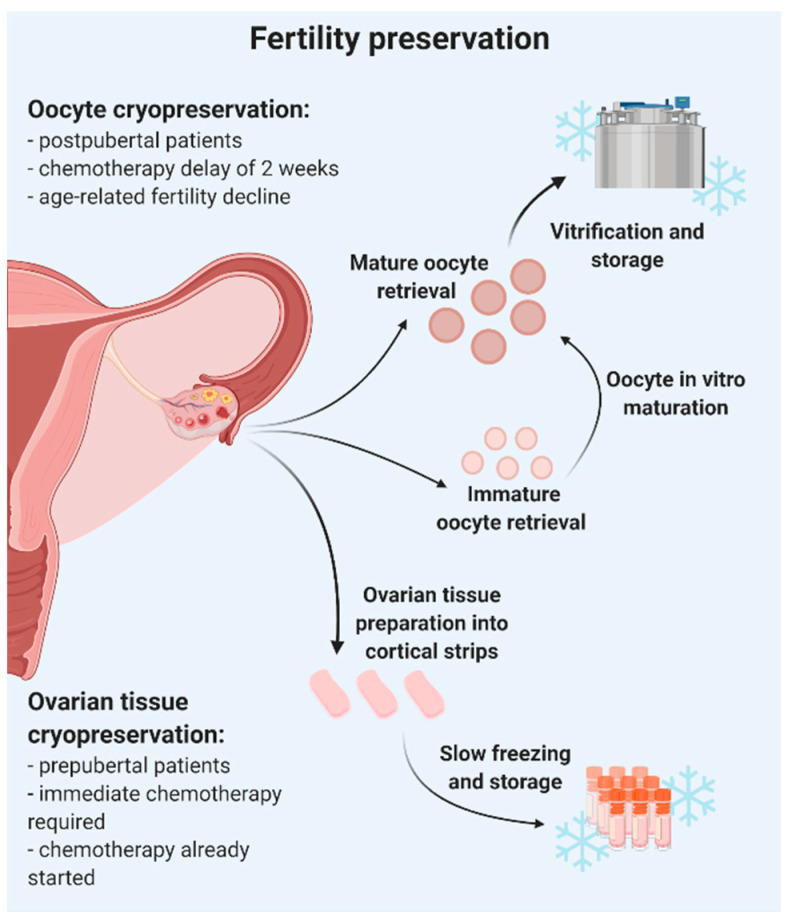
Oocyte and ovarian tissue cryopreservation as fertility preservation options. Created with BioRender.com (accessed on 23 August 2021).

Individuals presenting with benign disease or age-related fertility decline do not have the same pressing need as cancer patients. The choice of fertility preservation technique is mainly dictated by the age and pubertal status of the patient, and personal and medical circumstances. Oocyte vitrification yields the best results in adult women with benign diseases, those who wish to preserve their fertility for personal reasons and cancer patients if their therapy can be put on hold; and OTC is best for prepubertal girls and women whose treatment cannot be postponed (Figure 1). Both fertility preservation techniques and results are discussed in this review according to age.

## 2. Embryo and Oocyte Cryopreservation

Embryo cryopreservation is an effective technique, but a male partner is needed. This can raise ethical and legal concerns about potential orphan embryos if the patient dies or separates from her partner. Of all fertility preservation strategies, oocyte cryopreservation is the preferred option for postpubertal patients. It is a valid alternative for adult cancer patients, since it guarantees the woman’s autonomy [10]. Moreover, the technique of oocyte vitrification may ensure very high survival rates after warming [11].

Although some authors have reported an impaired ovarian response to controlled stimulation in cancer patients, it remains contentious, and appears to primarily affect specific cancers, such as ovarian malignancies [12] or BRCA mutations [13]. Indeed, recent studies in cancer patients do not confirm this apparently impaired reaction [14].

Before contemplating embryo or oocyte cryopreservation in women with cancer, it must be determined that the fertility preservation option will not delay the start of chemotherapy. While ovarian stimulation can be initiated at any time during the menstrual cycle [15], and luteal phase stimulation is possible with equivalent results in terms of obtained mature oocytes, it is important to leave at least 10 days before the start of the chemotherapy. This random-start ovarian stimulation protocol is now routinely applied for fertility preservation in cancer patients [16].

The success of the technique and live birth rates show strong correlations with age at the time of oocyte cryopreservation, with 35 years appearing to be the cut-off. Indeed, even with the same number of frozen oocytes, the likelihood of a live birth in patients aged over 35 years at the time of oocyte vitrification is significantly diminished [17,18].

Apart from age, the other determinant of cumulative live birth rates is the number of retrieved oocytes, with an ideal range of 10–15. In cancer patients, the quality of frozen oocytes appears to be equivalent to those cryopreserved for age-related fertility decline, but the number of oocytes that these patients can supply is limited due to the need to start gonadotoxic treatment without delay [14]. Since the number of frozen oocytes dictates the later chances of conception, it is vital that the maximum number of oocytes are frozen in the available time. Protocols have even been developed for double stimulation (follicular and luteal phase ovarian stimulation) during the same menstrual cycle, known as DuoStim [19]. The goal is to maximize the number of gametes obtained, improving cumulative live birth rates without postponing cancer therapy [20,21,22].

### 2.1. Disease-Specific Limitations

It is commonly accepted that alternative controlled ovarian stimulation (COS) protocols should be applied according to the steroid sensitivity of the cancer in question. Indeed, in women with estrogen receptor-positive breast cancer, tamoxifen or letrozole are used in addition to COS to theoretically inhibit breast cancer growth during stimulation [23]. However, no randomized controlled trials support the notion that these alternative COS schedules are superior to standard COS [23]. Recent studies have shown that there may even be a negative effect of letrozole or tamoxifen on fertilization and embryo quality in fertility preservation cycles [24].

One specific benign indication, namely, endometriosis, is somewhat set apart, since the number and quality of the eggs impair the final outcome, hence the cumulative live birth rate [25]. This confirms a detrimental effect of the disease itself on the ovarian reserve, as a detrimental effect of previous ovarian surgery.

### 2.2. In Vitro Maturation of Oocytes

The goal of obtaining mature metaphase II (MII) oocytes following COS is not always achieved. Studies have been conducted to assess whether oocytes that have been retrieved at immature stages (germinal vesicle (GV) or metaphase I (MI)) can be matured in the IVF laboratory [26]. This process is known as in vitro maturation and has been shown to enhance oocyte and embryo yields in breast cancer patients undergoing COS for fertility preservation purposes [27]. An innovative procedure was recently reported in a woman with breast cancer who did not undergo COS. Follicles were aspirated in the course of the natural cycle, and the oocytes obtained were therefore immature and subjected to in vitro maturation and vitrification. Thereafter, the oocytes were thawed and fertilized, resulting in embryo transfer and a live birth [28]. This technique does not delay the start of chemotherapy and can be applied in patients in whom the use of gonadotropins is contraindicated.

### 2.3. Combined Ovarian Stimulation and Removal of Ovarian Tissue

Ovarian stimulation with a view to freezing mature oocytes or embryos can be combined with OTC in order to increase the chances of success of fertility restoration after highly gonadotoxic treatment. In such cases, OTC should be performed before COS and oocyte vitrification, as this avoids having to cryopreserve ovarian tissue from blown-up cortex with bleeding corporea lutea. Moreover, freezing tissue before oocyte retrieval does not affect the mean number of retrieved MII oocytes, nor the quality of resulting embryos [29,30]. Nevertheless, COS can be started 1–2 days before OTC. The aim of initiating stimulation as soon as possible is to gain time, which is very precious in patients suffering from malignant diseases.

## 3. Ovarian Tissue Cryopreservation

OTC is the only available option for fertility preservation in prepubertal patients and adult women in whom gonadotoxic therapy cannot be postponed [1,31,32]. It has the advantage of preserving a large number of follicles at once and does not require any hormonal stimulation. Nonetheless, strict selection criteria must be applied in order to make the most of this technique [33,34].

### 3.1. Selection Criteria

Patient age at cryopreservation is of utmost importance, since reproductive outcomes are known to decline with age [35]. This was confirmed in a recent study by five leading European centers reporting a series of 285 women who underwent transplantation of frozen-thawed ovarian tissue [36]. They showed that the mean age at cryopreservation in women giving birth after reimplantation of their ovarian tissue (26.9 ± 0.7 years) was significantly lower than in those who did not give birth (29.8 ± 0.4 years). The upper age limit for OTC has been reasonably set at 35 years of age, when the ovarian reserve is still relatively abundant [1,33].

Another crucial criterion to examine is the risk of POI, which should be at least 50%. The risk of developing POI is challenging to predict, as it depends on the type and intensity of treatment received (alkylating agents being the most toxic), and the existing ovarian reserve [32,34,37]. It is therefore generally recommended that ovarian tissue be cryopreserved prior to initiation of chemotherapy in patients over 15 years of age [33]. Nevertheless, some clinical situations prove the exception to the rule, such as patients suffering from acute leukemia in whom OTC cannot be performed before the start of chemotherapy. In these circumstances, recommendations from the European Society of Human Reproduction and Embryology (ESHRE) state that, “Patients who have already received low gonadotoxic treatment or a previous course of chemotherapy can be offered OTC as a fertility preservation option” [38]. This is consistent with recent studies showing that exposure to chemotherapy before OTC does not alter the results of ovarian tissue transplantation in terms of live birth rates [36,37,39,40,41]. However, caution is advised when alkylating agents are administered prior to OTC. Several studies have demonstrated the harmful effects of these agents on reimplantation outcomes [36,37,42].

A further emerging criterion to consider before OTC and transplantation is pelvic radiotherapy. A recent report shed light on the impacts of pelvic radiotherapy on the outcomes of ovarian tissue transplantation [36], where out of 15 women who were given high pelvic radiation doses for anal or cervical cancer, not a single one gave birth. Undoubtedly, live birth rates after ovarian tissue grafting are inversely proportional to the pelvic irradiation dose received. This may be explained by poor vascularization in ovarian transplants due to a fibrotic reaction of irradiated pelvic tissue (including the peritoneum) and irradiation of the uterus [36]. Total body irradiation (12 Gy) in adults leads to a heightened threat of miscarriage, low birth weight and premature birth [43]. Uterine reproductive capacity is more sensitive to radiation during childhood, causing irreversible uterine injury with direct uterine radiation of >25 Gy, making pregnancy inadvisable [43]. In adults, irreversible damage is observed from 45 Gy, which contraindicates pregnancy [43]. Given the poor outcomes of ovarian tissue transplantation and obstetric risks associated with conditions requiring high doses of pelvic radiation (anal, rectal, cervical cancer), OTC and transplantation should be carefully contemplated before proceeding. Reimplanting ovarian tissue in patients who have undergone pelvic radiotherapy is possible, but the radiation dose and zone, along with the disease itself, are factors that must be considered in the decision-making process prior to transplantation.

### 3.2. Ovarian Tissue Retrieval and Freezing

The amount of tissue needed depends mainly on the risk of POI and the existing ovarian volume. Unilateral oophorectomy only precipitates menopause by 1 to 2 years [44,45], however, ovarian biopsies are often sufficient to preserve fertility [1]. Unilateral ovariectomy is usually performed when pelvic radiotherapy or total body irradiation is planned, and in very young girls, where the size of the ovaries is very small [46,47].

The most widely used cryopreservation technique is the slow-freezing protocol, which has led to almost all live births so far [39,46,48,49,50,51,52,53,54,55,56,57,58,59], except in 2 published cases, where both live births were obtained from vitrified ovarian tissue [60].

### 3.3. Immature Oocyte Collection during Ovarian Tissue Preparation

OTC can be coupled with collection of immature oocytes at the time of freezing. Immature oocytes can be isolated by puncture from visible antral follicles on the surface of the ovary [61], retrieved from the medium used for ovary preparation [62] or collected from surplus ovarian medullary tissue [63]. The immature oocytes are then matured in vitro, fertilized or not, depending on the partner status of the patient, and subsequently vitrified. Since the first live birth achieved from cryopreserved embryos issued from in vitro-matured oocytes in 2014 [64], there has been a growing interest in the technique [61,62]. Nevertheless, the success rate of in vitro maturation depends on the age of the patient. Indeed, successful in vitro maturation rates were shown to be significantly lower in prepubertal than postpubertal subjects (10.3% versus 28.1%, *p* = 0.002) [65]. This was confirmed by a second study where success rates hardly reached 15.5% in premenarchal compared to 28.2% in young postmenarchal patients [66]. Another very recent study demonstrated that in vitro maturation rates peaked at 38.3% between the ages of 18 and 24, but dropped dramatically in patients ≤5 or ≥30 years of age (4.6% and 8.9%, respectively) [62]. This approach represents an add-on method to potentially increase the fertility opportunities for cancer patients, especially in young women with cancer where transplantation of cortical tissue may pose a risk of relapse, but the in vitro maturation approach is currently too inefficient to be the only method used for fertility preservation [63].

### 3.4. Ovarian Tissue Reimplantation

**Figure 2:**
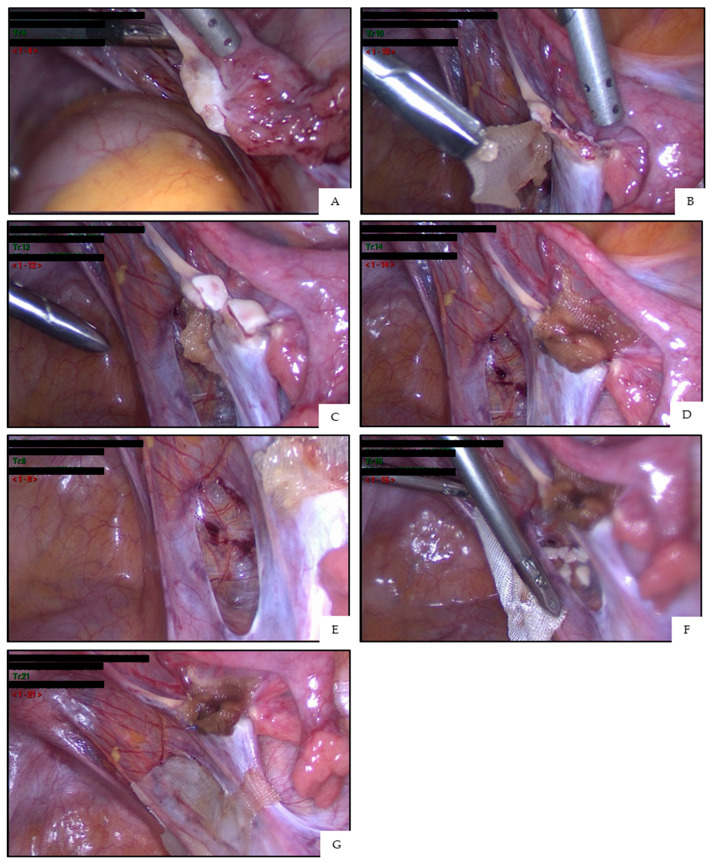
Orthotopic ovarian tissue transplantation in a patient aged 31 years, who underwent OTC prior to gonadotoxic chemotherapy (including cyclophosphamide) for stage IV Hodgkin’s lymphoma (according to the Ann Arbor classification). The procedure is performed by laparoscopy. Ovarian tissue transplantation onto the remaining ovary (**A**–**D**): the procedure starts by removing a large piece of ovarian cortex with scissors to expose the medulla with its vascular network, and Interceed^®^ is stitched to the inferior part of the ovary (**A**,**B**). Ovarian cortical pieces are then placed on the medulla and covered with Interceed^®^, the edges of which are fixed with fibrin glue (**C**,**D**). Ovarian tissue transplantation inside a peritoneal window (**E**–**G**): to create the peritoneal window, an incision is made on the anterior leaf of the broad ligament in a location where a vascular network is visible (retroperitoneal vessels) (**E**). The fragments are simply placed inside the window and covered with Interceed^®^, which is fixed with fibrin glue (**F**,**G**). (**G**) shows the result of the two orthotopic transplantation techniques performed side by side.

There are two ways of reimplanting ovarian tissue, namely, orthotopic transplantation (inside the peritoneal cavity onto the remaining ovary or into a specially created peritoneal window) and heterotopic transplantation (such as the forearm or abdominal muscle) [32] (Figure 2). Orthotopic transplantation is the procedure that has resulted in all live births to date, and grafting to the forearm has only produced one embryo [67]. If the goal of ovarian tissue transplantation is to achieve a live birth, orthotopic transplantation should be performed. However, if the aim is to restore natural hormone production, heterotopic transplantation is more convenient [68].

Recovery of ovarian function after transplantation takes 3.5 to 6.5 months, which is consistent with the time required for folliculogenesis to resume [31,69]. Ovarian function is restored in over 95% of cases, with an average graft lifespan of 4–5 years, and even up to 7 years in some cases [36,46].

Since the first live birth was achieved with this technique in 2004 [48], pregnancies and live births have continued to climb exponentially, reaching well over 200 by now [70]. Published live birth rates around the globe recorded figures of 31% in the Danish group in 2015 [71], 25% in the German FertiPROTEKT network in 2016 [56], 18.2% in the Spanish group in 2018 [72] and 41.6% in a Belgo-Israeli-American case series reported in 2020 [41]. Very recent data from five leading European centers involving 285 patients confirmed an overall pregnancy rate of 38% and a live birth rate of 26% [36].

**Figure 3:**
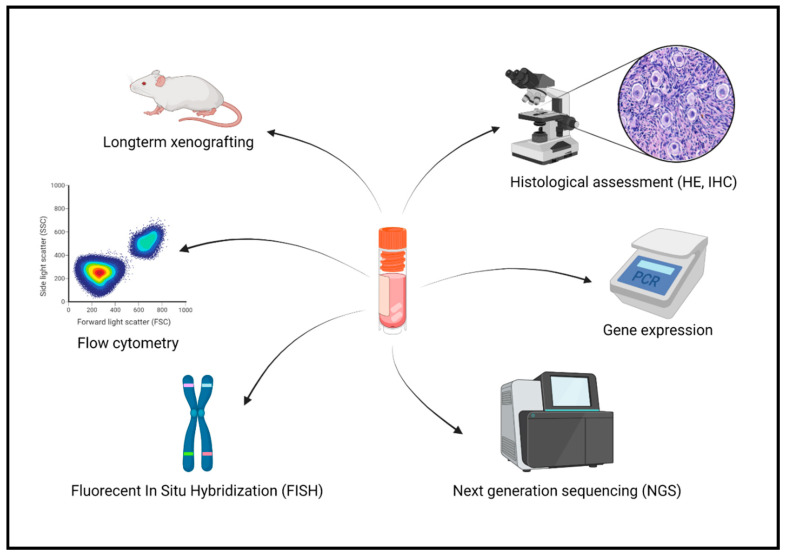
Screening of minimal disseminated disease in cryopreserved ovarian tissue. HE: hematoxylin and eosin staining; IHC: immunohistochemistry. Created with BioRender.com (accessed on 30 August 2021).

The main drawback of OTC is the risk of reimplanting malignant cells together with the ovarian tissue [73,74]. Earlier studies showed that the risk is high (>11%) in cases of leukemia, Burkitt lymphoma and neuroblastoma; moderate (0.2–11%) in cervical adenocarcinoma, advanced breast cancer, Ewing sarcoma and non-Hodgkin’s lymphoma; and low (<0.2%) in all other pathologies [73,75,76]. Inducing complete remission through several chemotherapy cycles prior to OTC in cases at high risk of ovarian contamination is reasonable, since it helps to reduce the risk of ovarian metastasis and does not interfere with reproductive outcomes after transplantation [36,37,40,77,78]. To the best of our knowledge, 4 live births have been reported to date in patients transplanted with ovarian tissue cryopreserved at the time of complete remission of their acute leukemia [79,80,81,82]. Nevertheless, excluding the presence of malignant cells by histological, immunohistochemical and molecular analysis was always performed and is highly recommended before contemplating reimplantation [79,80,81,82,83] (Figure 3).

## 4. How to Preserve Fertility in Children and Adolescents

Owing to improved survival rates in children and adolescents treated for cancer, there will be a growing population of adult survivors of childhood cancer seeking to have children. For this reason, there are concerns about fertility preservation even in children and adolescents. Moreover, indications for fertility preservation other than cancer are also increasing, since a number of non-malignant and chronic diseases require gonadotoxic treatment. It is also proposed in case of diseases associated with a premature decline of the ovarian follicle pool.

OTC and mature oocyte cryopreservation are the main techniques used for fertility preservation in children and adolescents. Although these two methods are validated in adults [84], these same fertility preservation techniques are still a challenge in children and adolescents. 

### 4.1. OTC in Children and Adolescents

This is the only fertility preservation approach that can be applied to girls before puberty and the one most commonly proposed to adolescents. Due to high follicular density in children and adolescents, it is only indicated in case of highly gonadotoxic treatments, including high-dose alkylating agents, pre-allograft and autologous hematopoietic stem cell conditioning, high-dose ovarian radiotherapy and gonadectomy. This strategy may also be implemented in case of non-malignant diseases when highly gonadotoxic therapy is needed, as in allografting of hematopoietic stem cells in sickle cell disease, or when fertility is likely to be prematurely impaired, as in case of Turner syndrome (TS).

The first report on OTC exclusively in children and adolescents was published in 2007 [85]. This was a series of 47 patients with a median age of 5 years (10 months to 15 years) at the time of cryopreservation and all were affected by oncological diseases. Since then, several studies on OTC in children and adolescents have been conducted (Table 2) [4,86,87,88,89,90,91,92,93,94,95,96,97,98,99,100].

Age for this approach varied between teams, ranging from 0.3 to 8 years [4,87]. Most patients were suffering from cancerous conditions, with the exception of one series, in which all the subjects had TS [87]. Among malignant diseases, the most frequent were found to be hematological malignancies (acute leukemia or lymphoma) [88,89,90,91,92,95,98,99,100], bone tumors [94], or specific diseases of childhood, such as neuroblastoma [4] or rhabdomyosarcoma [96].

Depending on the series, the percentage of non-malignant pathologies ranged between 5% and 33% [93,95,100]. The most common were TS [87,89,94] and hemoglobinopathies [4,95,96].

OTC can be coupled with freezing of oocytes isolated by puncture from visible antral follicles on the surface of the ovary or retrieved from the medium used for ovary preparation [101]. It was shown that it is possible to collect isolated oocytes even from very young girls [4], but successful in vitro maturation rates were shown to be halved in prepubertal compared to postpubertal subjects [[65,66], see paragraphe 3.3]. This technique does not cause any additional burden and the availability of isolated oocytes may be crucial, especially in case of leukemia where ovarian transplantation may be contraindicated [63].

### 4.2. Autotransplantation of Cryopreserved Ovarian Cortex from Patients Aged 18 and Under

In 2012, the functionality of cryopreserved ovarian tissue before menarche was demonstrated for the first time. Subcutaneous transplantation of ovarian tissue cryopreserved at the age of 10 years restored ovarian hormone function and induced puberty [102]. This result was confirmed the following year by another team [103].

Frozen-thawed ovarian cortex transplantation is currently the only technique that allows these patients to have children after ovarian tissue banking. Due to a lack of long-term experience and the relatively recent development of the technique, there are still very few data on outcomes of ovarian transplants performed with ovarian tissue from children or adolescents. Table 3 summarizes data on autografts of ovarian cortex cryopreserved before the age of 18 years with the goal of restoring fertility [4,39,40,52,80,82,86,98,104,105,106,107,108,109]. A total of 15 patients were involved. Nine patients had a malignant disease, and the remaining six had non-malignant conditions.

Five patients had not experienced menarche before OTC [4,39,107,109], and 8 patients had already had chemotherapy. After ovarian tissue retrieval, all patients underwent highly gonadotoxic treatment. In 12 subjects (80%), ovarian function resumed, including in 3 girls who were not pubertal at the time of OTC [4,107,109]. Of the 15 patients, 9 conceived at least once (60%) and 7 gave birth to at least one child (47%), including 2 who were not pubertal at the time of OTC [107], the youngest of whom was only 9 years old [109]. Although the number of patients is still small, rates of recovery of ovarian function, pregnancy and patients giving birth to at least one child are not lower than those obtained from ovarian tissue collected after the age of 18.

### 4.3. Mature Oocyte Cryopreservation after Controlled Ovarian Stimulation

Post-menarche adolescents may also benefit from mature oocyte freezing. While medically possible, mature oocyte cryopreservation has limitations in adolescents due to the invasive nature of the procedure. This technique should be proposed, depending on the patient’s maturity and virginity status.

Rare cases of ovarian stimulation have been reported in patients under 18 years of age (Table 4) [110,111,112,113,114,115,116,117]. Two patients had not reached menarche by the time of ovarian stimulation [111,116], one of whom was just 7 years of age [116]. In the cases shown in Table 4, diseases which were most prevalent were non-malignant, such as TS [112,117] and sickle cell anemia [113,114]. The number of oocytes retrieved ranged from 4 to 31 [117], and the number of cryopreserved oocytes ranged from 1 to 30 [113]. Despite the issue of virginity, oocytes were collected by a transvaginal procedure in most cases. In a girl aged 7 at the time of ovarian stimulation, oocyte retrieval was done transabdominally. Larger series reporting oocyte cryopreservation in cancer patients have shown that numbers of oocytes collected and numbers of mature oocytes obtained did not differ between patients under 20 years of age and patients over 20 years of age [118], nor between patients aged 13 to 17 years and those aged 18 to 21 years [117]. By contrast, cancellation rates were higher in patients under 20 years of age compared to patients over 20 years of age (10% vs. 6.6%) [118], and as high as 21% in girls aged under 18 years of age [97]. This increased cancellation rate might be attributed to a number of factors that are not necessarily related to a poor ovarian response. Physicians are naturally cautious to avoid complications in adolescents and, given their peripubertal status and maturation degree of folliculogenesis, are aware that possible differences in oocyte maturity and development could impair the response to gonadotropin stimulation [113,118,119]. Moreover, the vast majority of young subjects undergoing COS fall into a specific category of patients who will be or have been exposed to gonadotoxic therapies. Previous studies have revealed lower oocyte yields in oncology patients prior to treatment, likely due to use of lower gonadotropin doses [120,121]. In addition, the ovarian response might be attenuated in cancer patients due to malignancy-induced suppression of the hypothalamic-pituitary-gonadal axis or production of gonadotoxic cytokines [122,123]. Whether or not young patients require higher doses of gonadotropins needs further investigation, but an adolescent’s best interests in terms of efficiency and well-being are paramount.

### 4.4. Use of Cryopreserved Oocytes before the Age of 18

To our knowledge, only the birth of one boy has been reported after the use of oocytes cryopreserved before the age of 18. This was a patient who had 14 oocytes collected and frozen at the age of 17 in the context of a non-malignant pathology [110]. As many publications report lower oocyte quality in adolescents [124], it is important to follow up on outcomes of cryopreserved mature oocyte use in adolescents, to determine whether this technique could be routinely used in this specific population.

### 4.5. Fertility Preservation in Turner Syndrome

**Figure 4:**
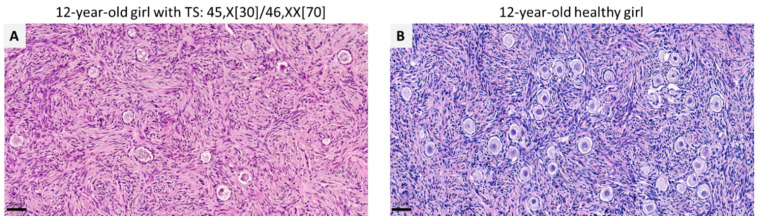
Representative histological sections of ovarian tissue from a 12-year-old girl with mosaic TS (**A**) and a healthy girl of the same age (**B**). Follicle density in ovarian tissue from the TS girl (**A**) was 872.34 follicles/mm^3^, and that of her healthy counterpart (**B**) was 2172.9 follicles/mm^3^. Scale bar: 50 µm.

TS, a common chromosomal aberration characterized by total or partial loss of one of the X chromosomes, occurs in 1/2000 to 1/2500 live-born females [125,126], but up to 90% of Turner cases result in miscarriage [127], making its fetal incidence much higher. POI is one of the major concerns for women with TS and their parents. Due to accelerated follicle atresia in TS patients, 80% of adolescent girls undergo POI prior to or around the time of puberty [128], leading to diminution or complete loss of their fertility potential (Figure 4). To address this issue, fertility preservation is indicated in TS patients who wish to have their own genetic offspring in the future by means of oocyte cryopreservation in postpubertal girls, and OTC in prepubertal girls [129,130].

The overall incidence of spontaneous puberty in TS is reported to be 5–20% [131,132,133,134]. Mature oocyte vitrification for fertility preservation purposes has been reported in postpubertal girls and adults with TS (age range: 13–28 years) [112,135,136,137,138]. Among these cases, only 2 patients had complete 45,X monosomy, and the remaining subjects were diagnosed with mosaic TS by karyotyping of lymphocytes. Ovarian stimulation resulting in 6 MII oocytes for freezing has also been reported in a 7-year-old girl with mosaic TS despite the inactive hypothalamic-pituitary-ovarian axis [116]. Yields of MII oocytes for freezing appear to be lower in TS girls than in their normal karyotype counterparts. Regarding oocyte quality, recovering a significantly higher number of oocytes than in healthy women would probably make sense, but it requires several stimulation cycles, making it difficult to do in adolescents. Although mature oocyte cryopreservation in TS patients is feasible in practice, there is some uncertainty about this approach. It is unknown whether cryopreserved oocytes from TS girls will lead to successful embryo development or pregnancy. Despite some groups supporting the safety and efficacy of ovarian stimulation in children [112], oocyte pick-up procedures may be technically challenging in these young girls.

Due to various limitations of oocyte freezing, there is no alternative way of protecting future fertility other than OTC in many countries. The possibility of preserving fertility is strongly related to the number and quality of follicles residing within ovarian tissue [139]. A recently published retrospective case-control study of 15 girls and young women aged 5–22 years with TS found evidence of follicles in 60% of biopsies, albeit with a high rate of abnormal morphology [140]. Karyotyping of ovarian cells of small follicles from 5 TS patients also revealed high levels of aneuploidy in granulosa cells, which may have a negative impact on the development of follicles, although the majority of oocytes did show normal X chromosome content [141]. These findings suggest that the benefits of OTC and transplantation may be limited to a highly select group of mosaic TS patients. The first case of OTC in a young girl with mosaic TS was reported in 2008 [142]. This technique is already routinely offered to TS subjects in several countries, but its efficacy remains unknown in this population due to a lack of follow-up data. Indeed, there are no reports of children born to women with TS after autotransplantation of their ovarian tissue [143].

In summary, the risks of POI and infertility in TS patients are significantly high. To maximize the benefits of fertility preservation, TS girls and their parents should be informed and evaluated as soon as possible in childhood, since the ovarian reserve of the vast majority of TS patients may be depleted before adulthood. It appears that indicating OTC in TS girls too soon may be too aggressive, but waiting longer for oocyte cryopreservation may be too late and increase the risk of failure. Choosing the appropriate time for fertility preservation in TS patients remains a challenge that needs regular follow-up and careful analysis.

## 5. Conclusions

Patients of all ages should be counseled on the risk of iatrogenic POI at the earliest opportunity, and referred to fertility specialists to discuss existing fertility preservation options. Oocyte cryopreservation can be proposed to postpubertal patients when the start of chemotherapy can be postponed by at least 10–12 days. Cumulative live birth rates using cryopreserved oocytes for oncological reasons depend on the age at cryopreservation and the number of oocyte retrieved, which may be limited by the urgent need to initiate gonadotoxic treatment. Regarding oocyte freezing in prepubertal patients, the only case of use reported in the literature does not allow us to draw any conclusions on its effectiveness in this specific population.

OTC can be proposed to both pre- and postpubertal patients and when immediate chemotherapy is required or has already started. Transplantation ensures long-term endocrine function resumption and favorable fertility restoration rates.

In contrast to data obtained in adult patients, use of oocytes or ovarian cortex cryopreserved in childhood or adolescence is still limited due to the young age of patients at the time of fertility preservation. Indeed, most of them are not even of childbearing age yet. What is encouraging, however, is that initial results on use of cryopreserved ovarian tissue in this population do not appear to be inferior to those reported in adult women. 

## Figures and Tables

**Table 1:** Indications for fertility preservation.

**1. Non-oncological diseases** for which fertility preservation is indicated: ▪ Systemic diseases requiring chemotherapy, radiotherapy, and/or bone marrow transplantation ▪ Ovarian diseases: ○ Bilateral benign ovarian tumors ○ Risk of ovarian torsion ○ Severe and recurrent ovarian endometriosis ▪ Risk of premature ovarian insufficiency: ○ Turner syndrome ○ Family history
**2. Oncological diseases** requiring gonadotoxic chemotherapy and/or radiotherapy or bone marrow transplantation: ▪ Hematological diseases (leukemia, Hodgkin′s lymphoma, and non-Hodgkin’s lymphoma) ▪ Breast cancer ▪ Sarcoma ▪ Some pelvic cancers ▪ Central nervous system tumors
**3. Social reasons** ▪ Age ▪ Childbearing postponed to later in life

**Table 2:** Main series for ovarian cortex cryopreservation in children and adolescents reporting 25 or more than 25 patients (for teams publishing several series, only the most recent is considered here).

Authors (Year)	Patients	Age (Years)			Patients ≤ 12 Years Old	Patients ≤ 18 Years Old	Number of Oncological Diseases	Most Frequent Oncological Diseases	Number of Non-Oncological Diseases	Most Frequent Non-Oncological Diseases
	*n*	Mean	Median	Range	*n* (%)	*n* (%)	*n* (%)	(*n*)	*n* (%)	(*n*)
Borgström B et al. (2009) [87]	47	14.6	14.5	8–19.8	9 (19)	40 (85)	0	0	47 (100)	Turner syndrome (47)
Oktay K et al. (2009) [88]	26	14.3	16.5	4–21	9 (35)	21 (81)	18 (69)	AL (5)	9 (35)	Turner syndrome (2) Thalassemia major (2)
Jadoul P et al. (2010) [89]	58	10.4	11.6	0.8–15.8	32 (56)	58 (100)	48 (83)	ALL (12), Non-Hodgkin disease (8)	10 (17)	Turner syndrome (2) Recurrent ovarian cysts (2) Torsion of the ovary (2) Sickle cell disease (2)
Fabbri R et al. (2012) [90]	45	13.4	14.4	1.6–17.9	12 (27)	45 (100)	39 (87)	Hodgkin lymphoma (16)	6 (13)	Thalassemia major (2) Aplastic anemia (2)
Lima M et al. (2014) [91]	54	13.4	NA	NA	NA	NA	51 (94)	Hodgkin lymphoma (20) Ewing’s sarcoma (10)	5 (9)	Thalassemia major (2) Bone marrow aplasia (2)
Biasin E et al. (2015) [92]	47	NA	13	2.7–20	24 (51)	NA	39 (83)	Leukemia (30)	10 (21)	Thalassemia major (7)
Abir R et al. (2016) [93]	42	12.3	14	2–18	16 (38)	42 (100)	40 (95)	Leukemia (11) Hodgkin lymphoma (10)	2 (5)	Thalassemia major (1) Lupus Nephritis (1)
Jensen AK et al. (2017) [94]	176	11.3	NA	0.6–17.1	32 (18)	176 (100)	154 (87)	Bone tumors * (31) Hodgkin lymphoma (25) ALL (21)	22 (13)	Turner syndrome (6) Galactosemia (4)
Armstrong AG et al. (2018) [95]	114	8.1	8	0.5–14.6	93 (81)	114 (100)	76 (67)	Leukemia/myeloproliferative/ myelodysplastic diseases (20)	38 (33)	Hemoglobinopathies (18) Aplastic anemia (6)
Rowell EE et al. (2019) [96]	64	NA	12	0.5–23	31 (48)	NA	53 (83)	Rhabdomyosarcoma (14) Ewing’s sarcoma (10)	11 (17)	Hematologic disorders (6) Disorder of sex development (5)
Poirot C et al. (2019) [4]	418	7.5	6.9	0.3–15	325 (78)	418 (100)	313 (75)	Neuroblastoma (93) AL (76)	105 (25)	Hemoglobinopathies (71) Immunodeficiency (19)
Rodriguez-Wallberg K et al. (2019) [97]	114	13.8	NA	3–17	48 (42)	114 (100)	NA	NA	NA	NA
Lotz L et al. (2020) [98]	102	14.8	NA	6–17	NA	102 (100)	81(79)	Lymphoma (34) Acute leukemia (13) Osteosarcoma (11)	21 (21)	Ovarian tumour (8) Turner syndrome (6)
Takae S et al. (2021) [99]	25	NA	13	1–17	9 before menarche (36)	16 (64)	18 (72)	Acute leukemia (8)	7 (28)	Aplastic anemia (2) CAEBV (2) CHAI (2)
Joshi VB et al. (2021) [100]	38	NA	11	0.83–17	20 prepubertal (53)	14 pubertal (37)	36 (95)	Muscoskeletal malignancies (18) Hematologic malignancies (12)	2 (5)	Genetic syndrome (1) Non-malignant hematologic condition (1)

* Bone tumors include Ewing’s sarcoma and osteosarcoma; NA: not available; ALL: acute lymphoblastic leukemia; AL: acute leukemia; CAEBV: chronic active Epstein-Barr virus infection; CHAI: CTLA-4 haploinsufficiency with autoimmune infiltration disease.

**Table 3:** Autologous transplantation of ovarian cortex tissue cryopreserved at the age of 18 years and under.

Authors (Year)	Diagnosis	Age at OTC (Year)	Menarche before OTC	Chemotherapy before OTC	Planned Treatment	Age at OTT	Ovarian Function Recovery	Pregnancies/Births
Donnez J et al. (2011) [104]	Neuroectodermal tumor	17	NA	Yes	HSCT	24	Yes	One birth (boy)
Stern C et al. (2011) [105]	NHD	17	Yes	Yes	AlloHSCT	27	Yes	Biochemical pregnancy
Donnez J et al. (2012) [52]	Ovarian abscesses	18	NA	No	Bilateral oophorectomy	26	Yes	One birth
Macklon KT et al. (2014) [106]	PNH	18	Yes	No	HSCT	22	Yes	One birth (boy)
Demeestere I et al. (2015) [107]	Sickle cell disease	13.9	No	Yes	HSCT	24	Yes	One birth
Meirow D et al. (2016) [39]	Ewing’s sarcoma	14	No	Yes	ND	21	No	/
Povoa A et al. (2016) [108]	Ovarian cysts	18	Yes	No	Unilateral oophorectomy	28	Yes	/
Silber SJ et al. (2018) [80]	Myeloproliferative disorder	18	NA	Yes	HSCT	25	Yes	Three births (1 boy, 2 girls)
Matthews SJ et al. (2018) [109]	Beta thalassemia	9	No	No	HSCT	23	Yes	One birth
Poirot C et al. (2019) [4]	Neuroblastoma Sickle cell disease	12 11.2	No * No *	Yes No	HSCT HSCT	24.7 * 28.3 *	No Yes *	No * No *
Poirot C et al. (2019) [40]	NHD Shwachman-Diamond syndrome	16.6 16.1	Yes * Yes *	Yes No	HSCT HSCT	28.7 28.3	Yes No	One birth /
Lotz L et al. (2020) [98]	HL	17	NA	No	Chemotherapy for HL	32	Yes	No
Rodriguez-Wallberg KA et al. (2021) [82]	ALL	14	NA	Yes	AlloHSCT	29	Yes	Ongoing pregnancy

* Additional information provided by C Poirot. HL: Hodgkin lymphoma; NHD: non-Hodgkin disease; PNH: paroxysmal nocturnal hemoglobinuria; HSCT: hematopoietic stem cell transplantation; HD: high dose; ALL: acute lymphoblastic leukemia; OTC: ovarian tissue cryopreservation; OTT: ovarian tissue transplantation; NA: Not available.

**Table 4:** Documented cases of controlled ovarian stimulation done for fertility preservation in patients ≤18 years of age.

Authors (Year)	Patients (*n*)	Age at COS (Years)	Menarche (Age)	Disease	Oocyte Retrieval Modalities	Number of Retrieved Oocytes	Number of Cryopreserved Oocytes
Kim TJ et al. (2011) [110]	1	17	NA	Non-malignant disease *	NA	14	14
Reichman DE et al. (2012) [111]	1	13	No	Myelodysplastic syndrome	Transvaginal	20 (8 M2,9 M1, 2 GV)	18
Oktay K et al. (2014) [112]	5	13 14 13 15 14	Yes Yes Yes Yes Yes	Turner syndrome Turner syndrome Turner syndrome Germ cell tumor ALL	Transvaginal Transvaginal Transvaginal Transvaginal Transvaginal	19 11/7 16 8 21	10 8/4 12 4 11
Lavery SA et al. (2016) [113]	8	14 15 16 16 16 17 18 18	Yes Yes Yes Yes Yes Yes Yes Yes	Sickle cell anemia Sickle cell anemia Sickle cell anemia Sickle cell anemia Sickle cell anemia Sickle cell anemia Sickle cell anemia Sickle cell anemia	Transvaginal Transvaginal Transvaginal Transvaginal Transvaginal Transvaginal Transvaginal Transvaginal	7 5 21 29 14 5 31 7	7 4 16 25 11 3 30 1
Pecker LH et al. (2018) [114]	1	15	NA	Sickle Cell anemia	Transabdominal	14 **	12
Garg D et al. (2019) [115]	1	14	Yes (11 years)	Hodgkin lymphoma	Transvaginal	13	11
Azem F et al. (2020) [116]	1	7	No	Turner syndrome	Transabdominal	6	6
Manuel SL et al. (2020) [117]	16	15.6 (13.0–17.8)	Yes	Malignant and non-malignant diseases	Transvaginal	13 (4–31) median	11 (1–28) median

* Secondary pulmonary hypertension caused by transposition of great vessels, ** 2 COS because of no response to ovarian stimulation. COS: controlled ovarian stimulation; NA: Not available; ALL: Acute lymphoblastic leukemia.

## Data Availability

No new data were created or analyzed in this study. Data sharing is not applicable to this article.
